# Role of social benefits for future long-term sickness absence, disability pension and unemployment among individuals on sickness absence due to mental diagnoses: a competing risk approach

**DOI:** 10.1007/s00420-021-01825-5

**Published:** 2021-12-28

**Authors:** Annina Ropponen, Jurgita Narusyte, Mo Wang, Sanna Kärkkäinen, Lisa Mather, Victoria Blom, Gunnar Bergström, Pia Svedberg

**Affiliations:** 1 Division of Insurance Medicine, Department of Clinical Neuroscience, Karolinska Institutet, 171 77 Stockholm, Sweden; 2 Finnish Institute of Occupational Health, Helsinki, Finland; 3 Center of Epidemiology and Community Medicine, Stockholm County Council, Stockholm, Sweden; 4 Finnish Institute for Health and Welfare, Helsinki, Finland; 5 The Swedish School of Sport and Health Sciences, Stockholm, Sweden; 6 Unit of Intervention and Implementation Research for Worker Health, Institute of Environmental Medicine, Karolinska Institutet, Stockholm, Sweden; 7 Department of Occupational Health Sciences and Psychology, Centre for Musculoskeletal Research, University of Gävle, Gävle, Sweden

**Keywords:** Sick leave, Sickness absence, Disability pension, Unemployment, Mental diagnoses

## Abstract

**Purpose:**

To investigate associations between social benefits and disability pension (DP), long-term sickness absence (LTSA, ≥ 90 days), or unemployment among Swedish twins with sickness absence (SA) due to mental diagnoses.

**Methods:**

This population-based prospective twin study included register data on first incident SA spell (< 90 days) due to mental diagnoses (ICD 10 codes F00-F99) during the follow-up 2005–2016. SA < 90 days due to other diagnoses than mental diagnoses or any other social insurance benefit was identified for the preceding year of the first incident SA spell due to mental diagnoses (coded yes/no). Comparing those with any previous social benefits vs without, cumulative incidence curve to compare time to an event, and Cox proportional hazards models for cause-specific hazard ratios (HR, 95% confidence intervals, CI) treating first incident DP, LTSA and unemployment as competing risks were modeled.

**Results:**

During follow-up, 21 DP, 1619 LTSA, and 808 unemployment events took place. Compared to those without, those with at least one benefit had a higher risk for DP (HR 5.03; 95%CI 1.80, 14.01), LTSA (1.67; 1.50, 1.84) and unemployment (1.24; 1.03, 1.50). The cumulative incidence for DP was very low, < 1%, for LTSA 80% with any previous social benefits vs. 60% without, and for unemployment ≤ 5%.

**Conclusion:**

Social benefits received during the preceding year of SA due to mental diagnoses (< 90 days) predict DP, LTSA, and unemployment. Hence, previous social benefits may provide means for early identification of persons at risk for exit from labor market.

## Introduction

Mental disorders have become the leading causes of sickness absence (SA) in developed countries (Arends et al. 2014; OECD 2013). Another aspect related to mental disorders with or without SA is that adverse outcomes in terms of risk of long-term SA and permanent exclusion from the labor market (i.e. disability pension, DP), are higher than in somatic diseases (OECD and Union 2018). Reducing the extent of labor market exit due to SA and DP is highly prioritized in Sweden (Nilsson 2020; Ropponen et al. 2021). SA is not only a problem to individuals, but also to their families, employers and society in general. Overall, not only for SA, the costs for mental health problems are tremendous and have been estimated to be at least 4% of GDP within EU (OECD and Union 2018). For example, individuals with long-term SA, i.e. > 6 weeks as set being the limit in the systematic review (Dekkers-Sanchez et al. 2008) are less likely to return to work, and more likely to leave the labor market via early retirement or DP, even when controlling for morbidity (Dekkers-Sanchez et al. 2008; Norder et al. 2017; Ubalde-Lopez et al. 2017). Furthermore, those with mental disorders have higher risk of not being employed and poor mental health is also an important predictor of labor market non-participation (Fleischmann et al. 2018; Hakulinen et al. 2016; Mather et al. 2015; Prang et al. 2016; Topor et al. 2019). Although mild mental disorders or symptoms are associated with SA, most people with mild mental health problems still work, and are likely to benefit from working (Manty et al. 2017; Rantonen et al. 2017; Svedberg et al. 2018). Thus, SA/DP due to mental disorders are in increase and may also affect the labor market participation among unemployed (Stahmeyer et al. 2018). Hence, we need to know more about the consequences for the individuals on SA due to mental diagnoses in terms of labor market participation.

A meta-analysis showed that both contact with a medical specialist, but also earlier SA were associated with less likely return to work among those with mental disorders (Nigatu et al. 2017). Some indications exist that receiving social benefits including unemployment, SA, or temporary DP benefits or similar may predict exit from labor market due to permanent DP (Ropponen et al. 2014), but also affect return to work or other labor market participation indicators (Oyeflaten et al. 2014; Vaez et al. 2007) as shown in a recent Swedish study (Topor et al. 2019). Social benefits that are paid to cover income loss can be due to medical work incapacity (SA/DP) and therefore they might reflect morbidity. In Sweden, all residents aged 16–65 years and having income from work, unemployment benefits, or student benefits are eligible for the national SA insurance system if they are unable to work due to disease or injury. However, as social benefits can be due to other reasons such as unemployment or parental leave, in this study we focused only indicators of long-term problems related to exit from labor market (i.e. SA/DP). Until now, little is known on the role of social benefits while accounting for the competing events in the analysis of the risk of labor market exit. Sweden as all Nordic countries has rather generous welfare systems compared to many other countries. For example, in the Nordic welfare SA benefit is available for all at work but not limited to those at work since also people on unemployment benefits can receive SA benefits. A population-based study in Sweden would provide possibilities to investigate various exits from the labor market related to SA due to mental diagnoses with less bias due to the welfare system although the international comparison would be limited to Nordic countries.

Until now, relatively few studies have investigated consequences of SA due to mental diagnoses in terms of unemployment or DP (Norder et al. 2017; Ubalde-Lopez et al. 2017). Although a recent interest has been in studies of young adults (Di Thiene et al. 2019; Helgesson et al. 2018) and discordant twin pairs (Mather et al. 2019a) to identify factors predicting labor market outcomes among those with mental health problems, most studies have focused on adverse outcomes of SA in general (Bryngelson et al. 2013; Dorner et al. 2015; Gjesdal et al. 2009; Gjesdal et al. 2008). Another aspect meriting further studies is the competing risks approach—i.e. to shed light on alternative labor market exit reasons (Ervasti et al. 2019).

The purpose of this study was to investigate the associations of previous social benefits on various exit reasons from paid employment (that is, DP, long-term SA (≥ 90 days), or unemployment) utilizing the competing risk approach among individuals with SA due to mental diagnoses < 90 days in a large cohort of Swedish twins.

## Methods

Data from the population-based prospective Swedish Twin project Of Disability pension and Sickness absence (STODS) were used (Svedberg et al. 2010). STODS includes all twins that were born in Sweden between 1925 and 1990 (*n* = 119,907 individuals) identified in the Swedish Twin Registry (STR) (Lichtenstein et al. 2006; Svedberg et al. 2010). Of all twins, approximately one-third are monozygotic (MZ), one-third same-sex dizygotic (DZ), and one-third opposite-sex (OS) DZ twins (Lichtenstein et al. 2006). The base data consisted of all individuals in STODS that were alive and living in Sweden in 2005 and present in the Micro-Data for Analysis of the Social Insurance System (MiDAS) database from the National Social Insurance Agency.

### Sample

For this open cohort study, we first identified the first incident SA spell (< 90 days) due to mental diagnoses (International Classification of Diseases 10th Revision (ICD 10) codes F00-F99) during the follow-up from January 1st 2005 until to the end of 2016. The limit of < 90 days of SA due to mental diagnoses was set as limit to capture SA not yet very long term and that limit has been utilized in earlier studies of the MiDAS database (Gemes et al. 2019; Ropponen et al. 2020). January 1st 2005 was selected as a starting point, since the MiDAS database includes diagnoses for SA from 2005 onwards. At this step we excluded those having a SA spell ≥ 90 days due to mental diagnoses if that had occurred before SA spell < 90 days. Second, we restricted the sample to those at at risk of DP: younger than 65, not emigrated, not on old-age pension, nor on DP. The final study sample included 3755 twin individuals, whereof 72 MZ, and 43 DZ, and 69 OS twin pairs. The mean age at the baseline was 39.6 years for women (69% of the final sample) and 40.2 years for men (31%).

### Exposures

**Fig. 1:**
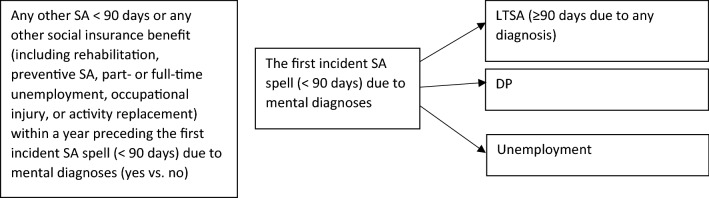
The analytic strategy among those who had the first incident SA spell (< 90 days) due to mental diagnoses since 2005. Censoring has not been accounted in this figure

SA < 90 days due to other diagnoses than mental diagnoses, or any other social insurance benefit targeted to support return to work (including rehabilitation, preventive SA, part- or full-time unemployment, occupational injury, or activity replacement) was identified the year immediately preceding the first incident SA spell of < 90 days due to mental diagnoses. Then we coded these earlier social benefits as yes/no for occurrence and used in the analyses as a predictor, i.e. any previous social benefit was a binary yes/no variable indicating at least one social benefit before SA spell < 90 days due to mental diagnoses (Fig. 1).

### Outcomes

Data on SA and DP benefits due to any diagnosis (date of beginning and end) paid by the Social Insurance Agency were collected from the MiDAS-database. The follow-up started from the end of the first incident SA (< 90 days) due to mental diagnosis when the individuals were not on SA, DP or unemployed. For DP, all incident cases were included. SA due to any diagnosis ≥ 90 days was included as an outcome (LTSA) (Fig. 1). Unemployment was assessed in Longitudinal Integration Database for Health Insurance and Labour Market Studies (LISA), Statistic Sweden, (Ludvigsson et al. 2019) by the amount of days being registered with the Swedish public employment service as a job seeker. Since the amount of days per year was the only available information from LISA (i.e. no starting date of unemployment), the follow up date was set as 1 January the year the unemployment occurred, and unemployment was coded as yes (≥ 1 day of unemployment) vs. no (no unemployment days).

### Social security in Sweden

All individuals in Sweden above the age of 16, with an income from work or unemployment benefits, can receive sick leave benefits paid by the Social Insurance Agency when disease or injury has caused reduced work capacity covering up to 80% of lost income. DP can be granted when that work incapacity is permanent and covers up to 65% of lost income. Employees receive sick pay from their employers during the first 14 days after a qualifying day without benefits (self-employed usually have more qualifying days). Information on the SA spells lasting ≤ 14 days was not available through the MiDAS. Unemployed individuals have one qualifying day and receive sick pay up to 80% of lost income from the Social Insurance Agency from the second day. SA and DP can be granted 25%, 50%, 75% or 100% of regular working hours and the net days are calculated taking part-time SA into account, i.e. two half SA days are calculated as one net day.

### Covariates and censoring

Information on covariates in 2005 including age, sex, family situation combining information on marital status and children living at home, type of living area, and years of education, and on censoring reasons including year of emigration and old-age retirement was obtained from the LISA register, Statistics Sweden. The covariates were selected based on the known associations with study outcomes (i.e. LTSA, DP and unemployment) (Oyeflaten et al. 2014; Samuelsson et al. 2012). Date of death (censored) were obtained from the National Board of Health and Welfare. Finally, all registry data were linked to the twin data using the unique ten-digit personal identification number assigned to all Swedish residents.

### Statistical analyses

We calculated descriptive statistics (frequencies and means) for the final sample. Then we modeled cumulative incidence curve (CIC) to compare time to an event modeling follow-up time in years (in this study to LTSA [≥ 90 days] or unemployment) for those with any previous social benefits and those without. CIC estimates the probability of ending up on an event between all the events of interest (= competing risks, i.e. DP, LTSA ≥ 90 days, and unemployment). The CIC estimates are shown as a cumulative incidence function (CIF) in Fig. 1.

Third, Cox proportional hazards models were run to investigate the association between any previous social benefits and incident DP, LTSA and unemployment for sub-distribution hazard ratios (SHR) and cause-specific hazard ratios (CSHR) with 95% confidence intervals (CI). The follow-up was until DP, LTSA or unemployment and calculated in days. Death, emigration, old-age pension, and age 65 were considered as censoring reasons, or the end of the follow-up (December 31, 2016), whichever came first. These hazard models treated DP, LTSA and unemployment as competing risks. Compared to the standard Cox proportional hazards models, where the follow-up of non-events terminates only due to censoring, competing risk analysis considers competing events that prevent the event of interest from occurring. Treating observations that end up on competing events as if they could later end up to the event of interest overestimates the probability of failure, and the bias is larger when the competition due to frequent competing events is heavier (Putter et al. 2007).

**Fig. 2:**
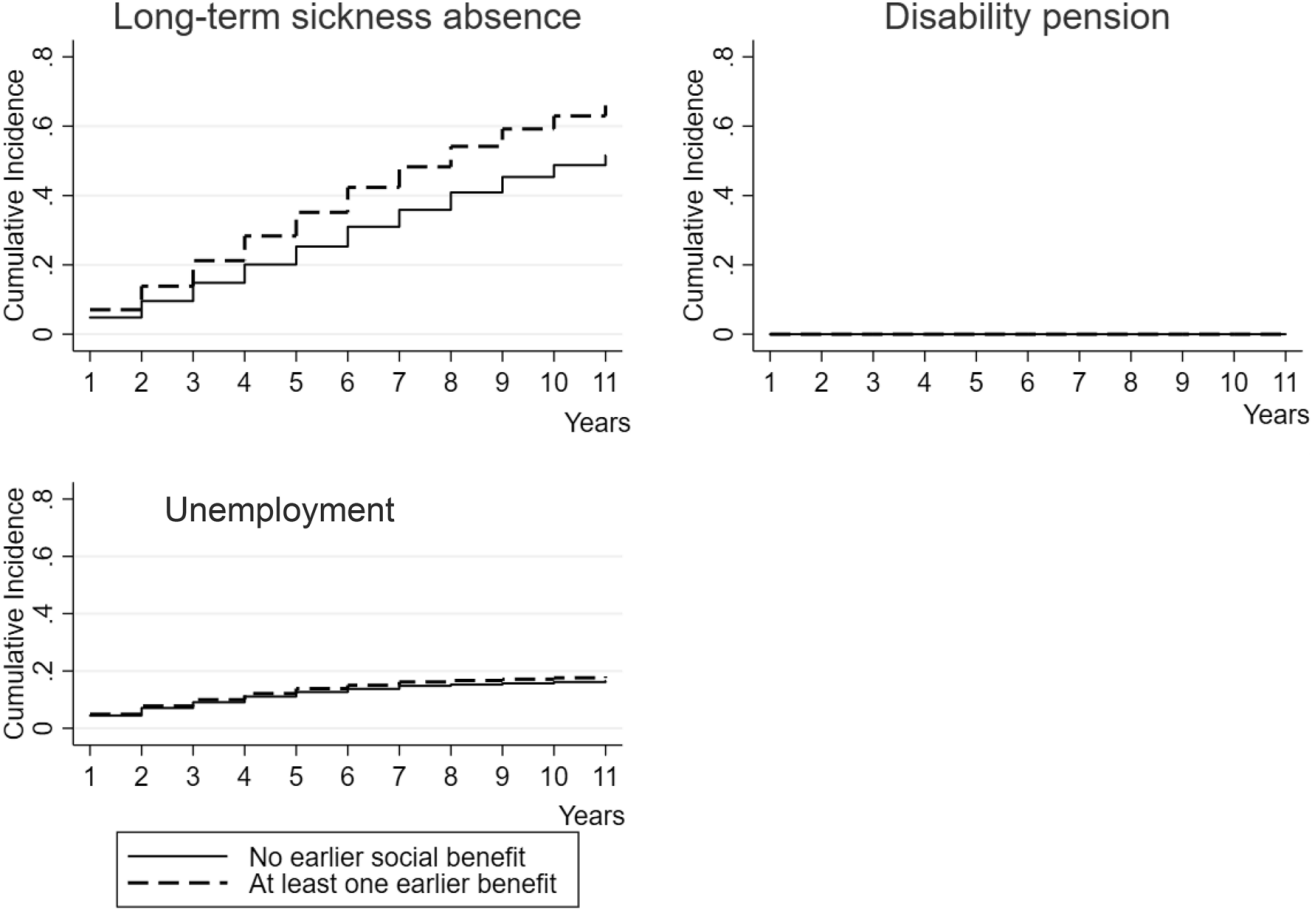
Cumulative incidence of long-term (> 90 days) sickness absence, disability pension or unemployment during the follow-up, stratified using the previous social benefits, among individuals with SA < 90 days due to mental diagnoses

The models were adjusted for covariates (age, sex, marital status, children living at home, type of living area, and years of education) and these were accounted in the CIFs (Fig. 2). Furthermore, we tested if zygosity played a role as this was a twin sample but adding zygosity to the models did not affect the point estimates, hence we chose not to report the results (data not shown). As we had very low number of complete twin pairs, we utilized the conditional Cox models for discordant pairs although the power was low and, therefore, the interpretation of the influence of familial factors, i.e. shared environment and genetics should be cautious.

All statistical analyses were performed with Stata MP 14.2.

## Results

The descriptive characteristics in 2005 are presented in Table 1. Among those with at least one previous benefit (39% of the final sample), the number of singles without children was a bit higher than among those with no benefits. The most common social benefit received before SA < 90 days due to mental diagnoses was unemployment benefits, then SA benefit < 90 days (due to any reason), and part-time unemployment (Table 1). The main diagnoses for SA < 90 days at two-digit ICD10-level were the F43 (reaction to severe stress and adjustment disorders) 51%, F32 (depression) 27%, F41 (other anxiety) 10% and F33 (recurrent depressive disorder) 3%, and altogether 39 different diagnoses were registered for the final sample. The mean follow-up time was 4.5 years (SD 3.3) for the whole final sample, 1.8 years (SD 2.1) for DP, 2.4 years (SD 2.4) for unemployment, and 3.6 years (SD 2.9) for LTSA.

**Table 1:** Descriptive characteristics of the final sample (*n* = 3755) of individuals with sickness absence (SA) < 90 days due to mental diagnoses

	All (*n* = 3755)		No benefits (*n* = 2307)		At least one previous social benefit before SA due to mental diagnoses (*n* = 1448)	
	***n***	%	***n***	%	*N*	%
Reason for end of follow-up						
Old-age pension	370	13	314	19	56	5
Rehabilitation	32	1	5	0	27	2
Sickness absence* (≥ 90 days)	1619	57	935	58	684	55
Unemployment	808	28	350	22	458	37
Disability pension	21	1	8	0	13	1
Occupational injury	1	0	0	–	1	0
Death	5	0	0	–	5	0
At least one previous social benefit (in the year before SA due to mental diagnoses)						
Preventive sickness absence					34	1
Unemployment					917	24
Part-time unemployment					340	9
Injury					11	0
Sickness absence^#^					566	15
Sex (women)	2594	69	1609	70	986	68
Education						
Low (≤ 9 years)	1257	33	867	38	390	27
Intermediate (10–12 years)	1927	51	1134	49	793	55
High (≥ 13 years)	566	15	303	13	263	18
Family situation						
Married or cohabitant without children	571	15	416	18	155	11
Married of cohabitant with children	1194	32	800	35	394	27
Single without children	1646	44	899	39	747	52
Single with children	344	9	192	8	152	10
Type of living area						
Big cities	1374	37	877	38	497	34
Medium-sized cities	1381	37	827	36	554	38
Rural areas	1000	27	603	26	397	27
	Mean	SD	Mean	SD	Mean	SD
Age (at 2005)	39.8	11.8	41.5	11.6	37.1	11.8

*Part-time and full-time combined, ^#^SA < 90 days due to any diagnoses

During follow-up, altogether 21 DP, 1619 LTSA, and 808 unemployment events took place. Compared to those without any previous social benefits, those with at least one benefit had a higher risk for DP as shown in Table 2 both for SHR and CSHR. The risk for LTSA and unemployment was also significant. These models were adjusted for age, sex, family situation, education, and type of living area. The conditional models indicated that for LTSA the risk remained but attenuated to statistical insignificance, whereas for unemployment, the risk was reversed although not significant. Although the power was low in these conditional models, they point that in unemployment we cannot rule out the effect of familial confounding.

**Table 2:** Hazard ratios (HR) with 95% confidence intervals (CI) from sub-distribution model (SHR), cause-specific hazards model (CSHR), and conditional Cox regression for discordant twin pairs

	Models accounting for covariates					
	**LTSA**					
	SHR	95%CI	CSHR	95%CI	HR*	95%CI
No early benefit	1		1		1	
Any early benefit	1.64	1.48, 1.84	1.67	1.50, 1.86	1.50	0.72, 3.11

	Models accounting for covariates					
	DP					
	SHR	95%CI	CSHR	95%CI	HR*	95%CI
No early benefit	1		1		1	
Any early benefit	4.77	1.72, 13.21	5.03	1.80, 14.02	–	–

	Models accounting for covariates					
	Unemployment					
	SHR	95%CI	CSHR	95%CI	HR*	95%CI
No early benefit	1		1		1	
Any early benefit	1.10	0.92, 1.33	1.24	1.03, 1.50	0.60	0.22, 1.65

*Conditional hazard ratio, i.e. comparison of discordant twin pairs

The cumulative incidence for DP among individuals with SA < 90 days due to mental diagnoses stratified by having previous social benefits or not was very low, i.e. < 1% with any previous social benefits ended up on DP during the 11-year follow-up, whereas 0% of those who had no previous social benefits (Fig. 2). The cumulative incidence function for LTSA among those without previous social benefits the rate was 60% and for those with at least one previous social benefit up to 80% during the follow-up. For unemployment, the corresponding rates were 4% and 5% (Fig. 2).

## Discussion

In this prospective study, we followed almost 4000 Swedish twin individuals with an incident SA spell < 90 days due to mental diagnoses and investigated the role of having had earlier social benefits on the risk of LTSA (≥ 90 days), DP or unemployment. Our results indicate that previous social benefits (i.e. < 90 days not due to mental diagnoses, rehabilitation, preventive SA, part- or full-time unemployment, occupational injury, or activity replacement in the year prior to the first SA spell < 90 days) increased the risk of LTSA, DP and unemployment. Results are in line with earlier findings (Ropponen et al. 2014; Vaez et al. 2007). As we utilized the competing risks approach, this study adds to the existing knowledge since the results indicated that the associations between previous social benefits and DP, LTSA, and unemployment exist even when comparing them to each other’s. This finding was further supported by the cumulative incidence of LTSA and unemployment which vary considerably. Hence, previous social benefits are associated with labour market exit but to different magnitude. These findings adds to the earlier studies that have mainly been cross-sectional (Armannsdottir et al. 2013), limited to one labor market exit only (Ropponen et al. 2014), utilized short follow-up (< 1 year) (Vogel et al. 2017), or focused on one occupational group (Stapelfeldt et al. 2014), to the few studies that have followed those on SA due to mental diagnoses (Helgesson et al. 2018) and to a study about elasticity of social insurance scheme for lost earnings due to SA (Böckerman et al. 2018).

The starting point for this study was the knowledge that (a) SA due to mental diagnoses are prevalent (Arends et al. 2014; OECD 2013), (b) exclusion from the labor market is higher among those with mental disorders compared to those with somatic diseases (OECD and Union 2018) and (c) most people with mild mental problems still work, and potentially also benefit from working (Manty et al. 2017; Rantonen et al. 2017; Svedberg et al. 2018). Our results of the associations between having had previous social benefits with labor market exits and of cumulative incidence among those with SA due to mental diagnoses seem to be affected by previous social benefits. Especially the almost non-existing difference in cumulative incidence of unemployment (along with non-significant SHR) between those with or without earlier social benefits points towards the importance of identification and support for those with SA due to mental diagnosis to promote timely return to work. Furthermore, the high cumulative incidence of LTSA in both groups (with and without earlier social benefits) emphasizes the need to consider even short SA due to mental diagnoses. Therefore, one may assume that any previous social benefit indicates actions taken by individuals. Hence, they should be identified and supported at the occupational health care for potential risks for exit from labor market or that societies and health care should provide some preventive means for support. One aspect meriting attention is the finding that the most common social benefit received before SA < 90 days due to mental diagnoses was unemployment benefits. This might reflect the higher level of unemployment among workers with mental disorders (Crespo-Facorro et al. 2021; Drake and Wallach 2020; McAlpine and Alang 2021), but further studies should address this in detail.

Although our results confirm the earlier findings that those on SA due to mental diagnoses are at risk of not returning to work, and more likely to leave the labor market via DP (Dekkers-Sanchez et al. 2008; Norder et al. 2017; Ubalde-Lopez et al. 2017), our findings related to LTSA and unemployment are new. In particular, the curves for cumulative incidence function indicate that social benefits might be more important for LTSA than unemployment based on relatively larger difference in incidence between receipt of social benefits vs. none and overall high incidence of LTSA. Another speculation is related to severity of diseases although our analyses could not shed light on that as we had ICD-10 codes at 2-digit level preventing from separating severe vs. mild diagnoses. Hence, this calls for workplaces, occupational health care and societies to react on any recipe of social benefits, and first incidence of SA due to mental diagnoses to support return to work and enhance possibilities to work even when work ability might be decreased due to mental ill-health. However, as we combined various social benefits together, one needs to bear in mind that although our results point to the direction that these benefits are associated with labor market exit and in line with earlier studies (Ropponen et al. 2014; Wikman et al. 2012), the results does not say anything about how effective these measures (including, for example, social benefits for rehabilitation and unemployment) are. Instead, these various social benefits were used as indicators of potentially more serious or lengthy problems.

This study had the benefit of utilizing national registry data, i.e. strengths include no reporting bias, no loss to follow-up and a prospective design with follow-up from 2005 to 2016. Furthermore, we had a large working age population-based sample which enabled us to identify almost 4000 first incident SA spells due to mental diagnoses to be followed for labor market outcomes. A limitation of this study is that we had only register data and therefore lacked access to many social, health related or lifestyle factors affecting employment o sustainable working life (Dorner et al. 2015; Mather et al. 2020; Mather et al. 2019a; Mather et al. 2019b). The utilization of register data, although from multiple sources, do not completely rule out common method bias (Podsakoff et al. 2012) which may have inflated our results, but one can assume that the direction of associations would be retained. Another limitation is that we had rather few complete twin pairs among those with SA due to mental diagnoses. But complete twin pairs enabled us to analyze the discordant twin pairs for the role of familial confounding, that is genetics and shared (mainly childhood) environment on associations between social benefits and LTSA, or unemployment, but not DP. Hence, for unemployment, we could not rule out familial confounding but we found no effect on the associations between social benefits and LTSA. Results should be confirmed in further studies and sex differences should also be tested. However, as shown in earlier studies of Swedish twins (i.e. based on partially the same dataset), the effects of familial confounding have been minor (Mather et al. 2019a; Ropponen et al. 2014). Still, one of the benefits of this study is that our findings apply to the general population since Swedish twins are representative for the general population (Zagai et al. 2019). Another limitation is the fact, that in real life, DP, LTSA and unemployment can also be recurrent, but this phenomenon was not accounted in our analyses being out of the scope of the current study but would merit further studies. Another real-life aspect meriting caution is age. Our sample of individuals with SA due to mental diagnoses < 90 days was around 40 years of age at 2005 when we initiated the follow-up. This means that perhaps the most severe cases of SA due to mental diagnoses or some cases with early occurring mental diagnoses have been underrepresented in this study. Yet another aspect related to our measure of SA is the fact that SA spells lasting ≤ 14 days were not available in MiDAS register data, although those with mental health issues utilize these first two weeks too, reimbursed by the employer. Further studies should elaborate also these shorter SA. Swedish twins represent the Swedish population in general well in terms of SA, DP and unemployment (Samuelsson et al. 2012; Zagai et al. 2019), but the generalizability of our findings to other countries except the Nordic with similar welfare model might be limited. Furthermore, a limitation is that we had not information about clustering the employees on various workplaces. That might have affected our results since the employees are not randomly assigned into workplaces. Further studies should take occupational sectors and workplace level into consideration.

To conclude, social benefits received during the preceding year of SA due to mental diagnoses (< 90 days) predict LTSA (≥ 90 days) and DP, but the role of previous social benefits is less clear for unemployment. Hence, both SA due to mental diagnoses, but earlier social benefits in specific, may provide means for early identification of persons at risk for labor market exit. From practical policy view, various social benefits are indicative for potentially more serious or lengthy problems.

## Data Availability

The datasets generated and analysed during the current study are not publicly available. According to the General Data Protection Regulation, The Swedish law SFS 2018:218, The Swedish Data Protection Act, the Swedish Ethical Review Act, and the Public Access to Information and Secrecy Act, these type of sensitive data can only be made available after legal review, for researchers who meet the criteria for access to this type of sensitive and confidential data. Readers may contact the last author regarding these details.
